# Patient satisfaction and loyalty in Japanese primary care: a cross-sectional study

**DOI:** 10.1186/s12913-021-06276-9

**Published:** 2021-03-25

**Authors:** Tsunetaka Kijima, Akira Matsushita, Kenju Akai, Tsuyoshi Hamano, Satoshi Takahashi, Kazushige Fujiwara, Yuko Fujiwara, Makoto Sato, Toru Nabika, Kristina Sundquist, Jan Sundquist, Yutaka Ishibashi, Shunichi Kumakura

**Affiliations:** 1 Department of General Medicine, Faculty of Medicine, Shimane University, 89-1, Enya-cho, Izumo-shi, Shimane 693-8501 Japan; 2 Nagi Family Clinic, Tsuyama Family Clinic, Yunogou Family Clinic, Family Practice Centre of Okayama, 292-1, Toyosawa, Nagi-cho, Katsuta-gun, Okayama, 708-1323 Japan; 3 Centre for Community-based Healthcare Research and Education, Shimane University, 89-1, Enya-cho, Izumo-shi, Shimane 693-8501 Japan; 4 Department of Sports Sociology and Health Sciences, Faculty of Sociology, Kyoto Sangyo University, Kamigamomotoyama, Kita-ku, Kyoto-shi, Kyoto, 603-8555 Japan; 5 Izumo Citizens Hospital, The Izumo Centre for Family Medicine, 1536-1, Enya-cho, Izumo-shi, Shimane 693-0021 Japan; 6 Omagari Clinic, The Izumo Centre for Family Medicine, 1941, Otsu-cho, Izumo-shi, Shimane 693-0011 Japan; 7 Hamada-shi Kokuho Yasaka clinic, Hamada-shi Kokuho Clinics, 530-1, I, Kitsuga, Yasaka-cho, Hamada-shi, Shimane 697-0027 Japan; 8 Department of Functional Pathology, Faculty of Medicine, Shimane University, 89-1, Enya-cho, Izumo-shi, Shimane 693-8501 Japan; 9 Center for Primary Health Care Research, Department of Clinical Science, Malmö, Lund University, Clinical Research Centre (CRC), Box 50332, SE-202 13 Malmö, Sweden; 10 Department of Medical Education and Research, Faculty of Medicine, Shimane University, 89-1, Enya-cho, Izumo-shi, Shimane 693-8501 Japan

**Keywords:** Patient loyalty, Patient satisfaction, Primary care, PCAT, First contact, Longitudinality, Family-centeredness

## Abstract

**Background:**

This study aimed to explore associations between various elements of primary care, patient satisfaction, and loyalty.

**Methods:**

This cross-sectional study used a modified version of the Primary Care Assessment Tool (PCAT), which was adapted for Japan. We distributed the PCAT questionnaire to patients aged 20 years or older at five rural primary care centres in Japan. We confirmed the validity and reliability of the measure for our study. Next, we examined which elements of primary care were related to patient satisfaction and loyalty using Spearman’s correlation and structural equation modelling.

**Results:**

Of 220 eligible patients, 206 participated in this study. We developed nine component scales: *first contact (regular access)*, *first contact (urgent access)*, *longitudinality*, *coordination*, *comprehensiveness (variety of care)*, *comprehensiveness (risk prevention)*, *comprehensiveness (health promotion)*, *family-centeredness*, and *community orientation*. *Longitudinality* and *first contact (urgent access)* were related with patient satisfaction. *Longitudinality*, *first contact (regular access)*, and *family-centeredness* were related to patient loyalty. In the structural equation modelling analysis, two variables were significantly related to loyalty, namely a combined variable including *longitudinality* and *first contact (regular access)*, along with *family-centeredness*.

**Conclusions:**

While a patient satisfaction model could not be distilled from the data, *longitudinality*, *first contact (urgent access)*, and *family-centeredness* were identified as important elements for the cultivation of patient loyalty. This implies that primary care providers need to develop a deep understanding of patients’ contexts and concerns and pay attention to their level of access to cultivate greater patient loyalty.

**Supplementary Information:**

The online version contains supplementary material available at 10.1186/s12913-021-06276-9.

## Background

Quality assessment provides direction to healthcare systems for quality improvement [1]. Patient experiences with primary care have multiple dimensions, including access, continuity, coordination of care, comprehensiveness, family-centeredness, and community orientation. Instruments such as the Primary Care Assessment Tool (PCAT), [2] Components of the Primary Care Index, [3] EUROPEP questionnaire, [4] General Practice Assessment Survey, (GPAS) [5] and General Practice Assessment Questionnaire (GPAQ), [6] have been developed to evaluate these primary care dimensions. Previous research on primary care demonstrates that each element of primary health care displays important roles and values. A study [7] conducted in 34 countries noted that more comprehensive, continuous, and accessible care in primary care was positively associated with patient experiences. In addition, better access to PCPs or having a regular physician with whom one has a personal relationship are related to fewer emergency department visits. A continuous doctor-patient relationship and a broad range of service from PCPs affects patient experience. Stroger community orientation for PCPs is related to offering a broader range of service, in particular, preventive care. As mentioned above, several effects of primary care components for patient experiences and patterns of heath care utilization were noted. Such components and patient experience were also affected by medical structure; capitation payment was positively related with patient experience; people with lower incomes postponed more primary care visits for financial reasons [7, 8].

In the Japanese health care system, health care facilities are paid through capitation. In the universal health insurance system, patients are required to pay 10–30% of medical fees based on their age. Low-income patients who need welfare assistance are exempted from payment. Japan also has free access, does not have a patient list system or registration system. Hence, patients can choose any medical facilities, irrespective of disease severity or insurance status [9]. For the Japanese primary care system, primary care has been provided by various specialists [10]. Japan is now developing the education system to include primary care as one of the new medical specialties. The Japan Primary Care Association (JPCA) has certified primary care specialists since 2010. The Japanese Medical Specialty Board established primary care specialist as one of the medical specialists in 2018 [10]. Those health care systems affect each primary care component; some patients have direct access to secondary/tertiary care facilities. Challenges in the integration of medical records or communication between medical facilities make continuity of care and coordination of care difficult. Several studies using questionnaires to assess primary care such as GPAS and PCAT generally employ patients’ overall perception of the quality of primary care [11–13]. Patient satisfaction was one of the important indices, and has been used to evaluate the quality of primary care and the access or the continuity of primary care providers (PCPs) [14–18]. However, some researchers have criticised patient satisfaction measurement as an indicator of healthcare quality [19, 20]. Their findings indicate that “intent to use the same facility again” is a more valid criterion than “satisfaction” [20, 21].

PCPs who develop trusting relationships with patients tend to foster deep loyalty, [22–25] facilitating continued service provision by the same PCPs [23]. Patient trust, a good physician-patient relationship, and patient satisfaction are strongly related to patient loyalty to PCPs [23]. Loyalty could be a criterion for care improvement, [22] but empirical evidence supporting this claim is limited. Regardless, it seems that patient loyalty is an important indicator of quality of care, alongside patient satisfaction. To explore associations between each primary care element with both patient satisfaction and loyalty, we employed a contextually-customised PCAT.

## Methods

A cross-sectional study was conducted to develop the questionnaire and examine associations between each primary care element with patient satisfaction and loyalty. To this end, we customised a version of the PCAT already adapted for Japan. This study was approved by the Shimane University Institutional Committee on Ethics. The questionnaire was anonymous, serving to protect the respondents’ confidentiality, even if they criticised their PCPs. Completing and returning the questionnaire after study explanation was considered informed consent.

### Development and adaptation of the questionnaire

A series of procedures was employed to adapt the PCAT for this study’s purposes. First, a native Japanese professional translator translated the PCAT into Japanese. Another expert translator then reviewed the Japanese PCAT version. Twelve primary care experts including family physicians, community health centre directors, healthcare research experts, and experienced primary care nurses reviewed the translated version using the Delphi procedure [26]. These experts evaluated each item’s necessity using a nine-point scale, were asked about necessary revisions and additions, and reached consensus on whether to alter, remove, or add items. Next, a focus group was conducted with a non-medical expert panel to confirm that the questionnaire was comprehensible to laypeople. The backward translation of the PCAT followed, conducted independently by two native English expert translators. The translators and health research experts jointly reviewed all items, discussed whether they were appropriately translated and raised any concerns. Finally, the questionnaire was adjusted to contain the most appropriate items, based on the expert reviews. Pilot testing was conducted with 35 patients in three local primary care centres in Japan, after which final revisions were performed.

### Satisfaction and loyalty

Respondents were asked about their overall satisfaction to assess the questionnaire’s criterion validity. Based on previous PCAT studies, [11, 12] we examined overall satisfaction using a five-point Likert scale (1 = *very dissatisfied*, 3 = *neutral*, 5 = *very satisfied*). We also assessed patient loyalty. In marketing studies, customer loyalty is defined as the intention to use the company or store repeatedly [27]. Accordingly, patient loyalty was defined as the intention to continue using the same provider or recommend this provider to others [28]. Recommending a service provider is understood as a particular outcome of patient loyalty, [23] as is consistency in the patient-physician relationship over time and the intention to return to the same provider in the future [22, 29]. Considering this, two items were viewed as indicators of loyalty: intended continuity (“Do you want to continue consulting the same physician from now on?”) and willingness to initiate contact (“Do you consult your physician first when you develop a new health problem?”). A four-point Likert scale (1 = *definitely not*, 2 = *probably not*, 3 = *probably*, 4 = *definitely*) was used. A neutral response (*Not sure/don’t remember)* was also provided. This scale was replicated in other questions.

### Data collection, survey for validation, and comparisons with each scale

We gathered homogeneous data from rural areas. Since patients’ experiences vary depending on where they live and on PCPs, we targeted similar low-population density areas rather than high-population density areas. The survey was conducted at primary care centres where physicians had been trained in family medicine. We defined a primary care centre as a facility providing family medicine practice, while also qualifying for residency training. Of 8 invited primary care centres, five such centres agreed to participate in this survey. The sample consisted of Japanese patients aged 20 years or older who had at least one consultation at the primary care centre. A research assistant informed patients of the study’s purpose and contents. Posting the completed questionnaire in the designated box was regarded as consent to participate. This survey was launched in May 2017 and lasted 3 months. Upon completion, respondents were given small gifts of appreciation, worth JPY 300.

We distributed the 67-item questionnaire, including questions about patient loyalty, overall satisfaction, the condition of primary care, medical expenses, health status, demographics, and regular healthcare providers. Regular healthcare provider was indicated through three questions adapted from the original PCAT: “*Do you have a regular doctor or medical facility that you go to when you do not feel well or would like a consultation about health issues?”*; “*Is there a doctor or medical facility that knows you well?”*; “*When you get medical care, do you have a doctor or medical facility that you trust?”* Patients who answered ‘No’ for all of the three questions were excluded. If they answered ‘Yes’ for at least one of the three questions, it was determined accurate that the doctor/facility they considered their PCP was in fact, their PCP and we had them complete the following questions.

### Statistical analyses

**Fig. 1:**
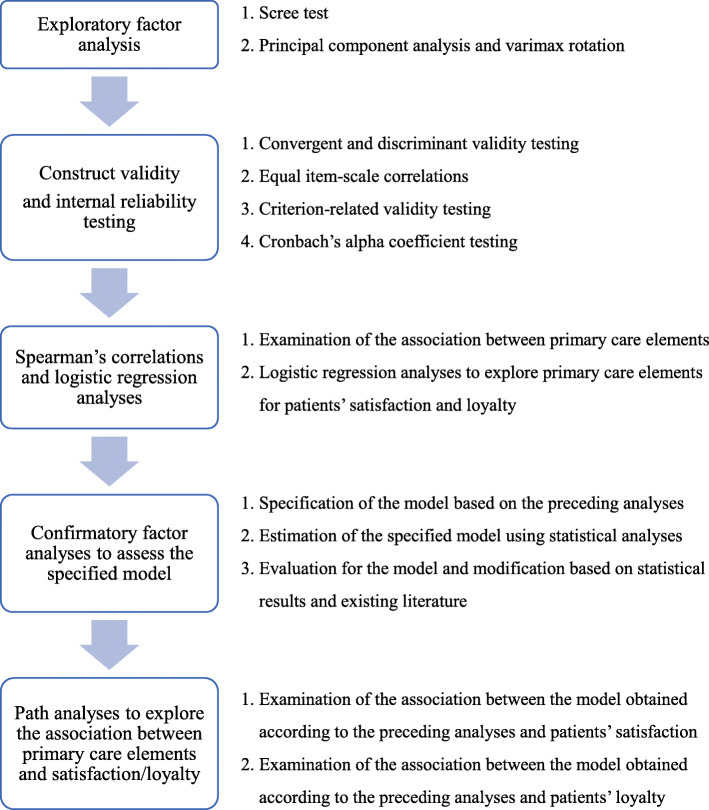
Flowchart of analyses

Data were analysed using JMP11, SPSS 22, and SPSS AMOS 25 software. We assigned a median value of 2.5 to *not sure/don’t remember* answers, consistent with previous PCAT studies [12, 30]. Likert scale values (1–4) were converted to scores between 0 and 100, with high scores indicating more favourable performance. Then, we performed the following procedures to create the structure of the items, to examine the questionnaire’s validity and reliability, and to explore the association between primary care element with patient satisfaction and loyalty (Flowchart of analyses in Fig. 1).

Exploratory factor analysis (EFA) examines various functions to reduce the number of variables, to inspect the structure or relationship between variables, and to evaluate the construct validity of a scale or instrument. Thereby, EFA enabled us to detect the main dimensions to generate a theory or model from a set of latent constructs represented by a group of question items [31].

First, we conducted EFA using principal component analysis and varimax rotation to examine construct validity. To determine the structure and the number of factors, we employed following criteria: factor loading of 0.36 or more and eigenvalues of 1.0 or more. Factor loadings of 0.36 or more were regarded as meaningful and used as a retention criterion [12].

Second, we examined construct validity, criterion validity, and internal reliability testing. Construct validity includes convergent validity and discriminant validity. Convergent validity was examined by confirming item-scale correlations; whether each item was correlated with the hypothesised scale. Item-scale correlations of 0.3 were defined as minimum acceptable scores and > 0.5 as favourable [30, 32]. Equal item-scale correlations were also measured using item-scale correlations, indicating that all items in a scale describe similar information about the concept. This was conducted to calculate the range of correlations obtained for all items in a scale, we defined its acceptable value as < 0.2 [32]. Discriminant validity was assessed by examining item-other scales correlations, and calculating scaling success; that is how often items within a particular scale correlated significantly more with their hypothesised scale than with any other scale [32]. Criterion validity testing was measured calculating correlations between total score and patient satisfaction/loyalty using Spearman’s correlation coefficient. We regarded correlations of > 0.30 as meaningful [33]. Internal reliability was assessed using Cronbach’s alpha coefficient where 0.70 was defined as the lowest acceptable value [12, 32]. We refined the validation of item placements in our final model by comparing whether its alpha when deleted was higher than the previously calculated alpha. Where this was the case, we considered removing the item to optimise our final model.

Furthermore, we examined the association between each scale score and patient satisfaction/loyalty by using Spearman’s correlations, and we explored the scale score for patient satisfaction/loyalty using multivariable logistic regression analysis. In addition, we conducted structural equation modelling (SEM) to confirm the developed questionnaire and to explore the scale score-patient satisfaction/loyalty associations. SEM includes confirmatory factor analysis (CFA) and path analysis. This analysis conducts the following procedure; (1) model specification/hypotheses (model identification), (2) model estimation, (3) model evaluation, (4) model modification [34]. This allows for evaluation of the specific set of assumptions developed based on the hypothesized model. SEM was employed to test our hypothesised theoretical model and to specify direct/indirect effects through a path model depicting the relationships among the observed variables [35]. This method was deemed appropriate considering the complex inter-relationships among primary care elements. For SEM, several assumptions must be met: no systematic missing data, sufficiently large sample size, multivariate normality, and correct model specification. Normality was assessed using skewness and kurtosis. We evaluated the specified model based on the results of previous statistical analyses and modified it according to statistical results and previously established methodologies.

The loyalty score was computed by calculating the mean score of two questions, one concerning intended continuity and the other willingness to initiate contact. Regarding SEM, we assessed the model fit for confirmatory factor analysis with the χ2 test, where *p* ≥ 0.05 supported appropriateness of fit, as well as by calculating the comparative fit index and the root mean square error of approximation (RMSEA).

## Results

We distributed 220 questionnaires, all of which were returned. However, 14 completed questionnaires were excluded as those respondents did not have a regular care provider; 206 completed questionnaires were therefore included. *Regular access and continuity* and *family-centeredness* were significantly related to loyalty in the SEM analysis. Table 1 shows the respondents’ demographics and extent of affiliation with a specific place/doctor. Patients’ mean age was 67.0 years. We conducted this study in three municipalities, where people aged 60–69 years accounted for most age groups in our targeted research population. Respondents in our study were also similar in all regions, although relatively older female respondents are highly represented in two areas compared with the reference population (Additional file 1). More than 95% of patients had been consulting their PCPs for one or more years.

**Table 1:** Characteristics of patients (*n* = 206)

Characteristics of patients	Number	%
Age		
Mean ± SD	67.0 ± 14.1	NA
range (year)	28–96	NA
missing data	20	
Sex		
Male	69	37.1
Female	117	62.9
missing data	20	
Occupations		
Employee/public servant	33	18.1
Employer	21	11.5
Student	0	0
Part time	23	12.6
Unemployed (including unemployed following retirement, and full-time homemaker)	83	45.6
Others	22	12.1
missing data	24	
Educational attainment		
Junior high school	41	23.0
High school, or high school equivalent	95	53.4
Professional trade school	22	12.4
University or graduate school	18	10.1
Others	2	1.1
missing data	28	
Self-reported Economic status		
high	131	74.0
low	46	26.0
missing data	29	
Health status		
Very good	9	4.8
Good	36	19.1
Fair	96	51.1
Not very good	40	21.3
Poor	7	3.7
missing data	18	
Number of primary care visits		
Once or less than 1 time/year	0	0
2-5times/year	22	10.9
6-9times/year	66	32.7
10times or more/year	114	56.4
Don’t know / Don’t remember	0	0
missing data	4	
The length of having been going to this provider		
Less than 6 months	3	1.5
From 6 months to 1 year	6	3
From 1 to 2 years	16	7.9
From 3 to 4 years	38	18.7
5 years or more	140	69
I don’t know / I don’t remember	0	0
missing data	3	

*SD* Standard deviation, *N/A* Not applicable

### Confirmatory factor analysis and validity

Sixty-seven items were analysed in the initial principal component analysis. For eigenvalues greater than 1.0, a seven-component solution was suggested (detailed scale in Additional files 2 and 3). All item factor loadings were equal to or greater than 0.36, and three items were excluded based on this criterion. Another item about a health problem survey was removed from the *first contact (urgent care)*, because we regarded it as unrelated to this component. One core component was divided into three components (*longitudinality*, *first contact (regular access)*, and *family-centeredness*) based on the primary care experts’ discussion regarding the component definitions. This was confirmed through validity and reliability testing. Upon assessing Cronbach’s alpha, one item was excluded from the *comprehensiveness (health promotion)* component, and two from *community orientation*, as they had a significantly lower alpha coefficient than the other items in each component. Following their exclusion, the Cronbach’s alpha of the scale increased significantly from 0.67 to 0.76 for *comprehensiveness (health promotion)* and from 0.67 to 0.75 for *community orientation*. Upon concluding the analysis, 60 items were included in the questionnaire (Additional file 2). The *first contact* component was further divided into two scales. *First contact (regular access)* relates to care received during office hours, whereas *first contact (urgent access)* relates mainly to consultation outside of office hours. The final version of the questionnaire entailed classifying the items according to nine component scales: *first contact (regular access)*, *first contact (urgent access)*, *longitudinality*, *coordination*, *comprehensiveness (variety of care)*, *comprehensiveness (risk prevention)*, *comprehensiveness (health promotion)*, *family-centeredness*, and *community orientation*.

Table 2 shows the results of convergent and discriminant validity, and internal reliability test outcomes. Convergent validity was favourable; all item-total correlations well exceeded the generally accepted minimum of 0.30 [32]. The average scaling success was 97.2% (525/540) for discriminant validity. Score reliability was sufficiently high (Cronbach’s alpha > 0.70). Table 3 presents additional descriptive features of the modified version of PCAT used in this study. For criterion validity, the total score was significantly related to patient satisfaction and loyalty (Spearman’s correlation coefficients were 0.40 and 0.55; both *p*-value < 0.01).

**Table 2:** Results of convergent and discriminant validity, and internal reliability (*n* = 206)

Scale	Number of items	Convergent validity	Discriminant validity			Internal reliability^b^
		Item-scale correlations	item-other scale correlations	Success/total	Scaling Success^a^ (%)	
First contact (regular access)	5	0.59–0.78	0.04–0.57	45/45	100	0.71
First contact (urgent access)	3	0.78–0.82	−0.33 - 0.22	27/27	100	0.75
Longitudinality	15	0.47–0.80	−0.09 - 0.67	121/135	89.6	0.91
Coordination	6	0.88–0.92	−0.10 - 0.51	54/54	100	0.95
Comprehensiveness (variety of care)	15	0.55–0.77	−0.08 - 0.62	134/135	99.3	0.93
Comprehensiveness (risk prevention)	7	0.65–0.84	0.02–0.56	63/63	100	0.91
Comprehensiveness (health promotion)	5	0.59–0.75	−0.07 - 0.54	45/45	100	0.76
Family centeredness	2	0.92–0.94	- 0.14 - 0.66	18/18	100	0.82
Community orientation	2	0.89–0.90	0.06–0.41	18/18	100	0.75
Total	60	NA	NA	NA	NA	0.95

^a^A scaling success rate was calculated as follows: a denominator is the total number of item-scale correlations tested, and a numerator is the number of those correlations for which the items in the scale correlate substantially greater with their own scale than with other scales.

^b^Cronbach’s alpha

NA: Not applicable

**Table 3:** Descriptive features of the modified PCAT (*n* = 206)

Scale	25th percentile	50th percentile	75th percentile	Range of score	Skewness	Kurtosis
First contact (regular access)	70.2	80.2	93.4	50–100	−0.12	−1.19
First contact (urgent access)	33.3	55.3	66.5	0–100	−0.18	−0.02
Longitudinality	69.2	82.4	93.7	39–100	−0.39	−0.80
Coordination	50.0	83.3	100	33–100	−0.27	−1.54
Comprehensiveness (variety of care)	54.5	66.8	76.0	4–100	−0.44	1.69
Comprehensiveness (risk prevention)	50.0	52.4	64.4	0–100	−0.67	1.78
Comprehensiveness (health promotion)	66.8	73.6	86.8	29–100	−0.18	0.01
Family centeredness	67	83.5	100	33–100	−0.37	−1.28
Community orientation	58.5	75	100	25–100	−0.07	−1.25
Total Score	64.0	70.8	81.0	45–98	0.23	−0.68

### Comparisons between each primary care element and patient satisfaction and loyalty

**Fig. 2:**
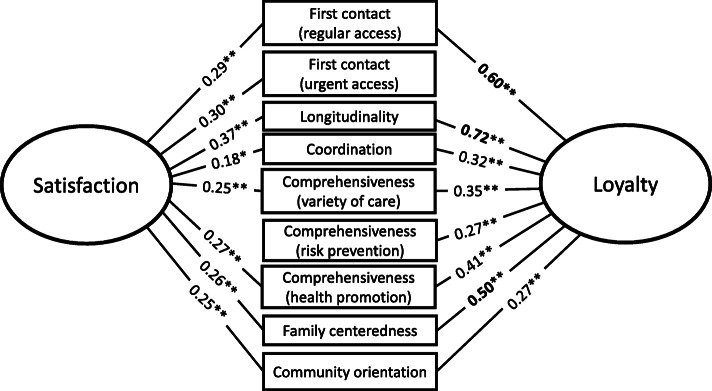
The associations between primary care each element, patient satisfaction and patient loyalty. * *P* < 0.05. ** *P* < 0.01

We assessed associations between all primary care scales with satisfaction and loyalty simultaneously. Table 4 shows a correlation matrix derived using Spearman’s correlations. *First contact (urgent access)* and *longitudinality* were significantly related to patient satisfaction. *First contact (regular access)*, *longitudinality*, and *family-centeredness* were significantly related to loyalty. Regarding the *first contact* element, *urgent access* was more strongly related to patient satisfaction than was *regular access* (Fig. 2). Though conducting multivariable logistic regression analysis, we did not fulfil the criteria for sufficient statistical analyses as most scale intercorrelations were high.

**Table 4:** Correlation Matrix of primary care scales, satisfaction, and loyalty (*n* = 206)

Variable	FC-r	FC-u	LG	CR	CO-v	CO-r	CO-h	FA	CM	To	Sa	Lo
FC-r	NA	0.15	0.66**	0.31**	0.42**	0.36**	0.39**	0.54**	0.35**	0.67**	0.29**	0.60**
FC-u		NA	−0.008	− 0.07	−0.16	− 0.15	−0.04	− 0.15	0.05	0.14	0.30**	−0.01
LG			NA	0.45**	0.56**	0.43**	0.44**	0.67**	0.31**	0.73**	0.37**	0.72**
CR				NA	0.39**	0.31**	0.26**	0.43**	0.28**	0.60**	0.18*	0.32**
CO-v					NA	0.63**	0.59**	0.61**	0.41**	0.76**	0.25**	0.35**
CO-r						NA	0.50**	0.50**	0.34**	0.67**	0.15	0.27**
CO-h							NA	0.49**	0.41**	0.68**	0.27**	0.41**
FA								NA	0.40**	0.76**	0.26**	0.50**
CM									NA	0.63**	0.25**	0.27**
To										NA	0.40**	0.55**
Sa											NA	0.26**
Lo												NA

*FC-s* First contact (regular access), *FC-u* First contact (urgent access), *LG* Longitudinality, *CR* Coordination, *CO-v* Comprehensiveness (variety of care), *CO-r* Comprehensiveness (risk prevention), *CO-h* Comprehensiveness (health promotion), *FA* Family centeredness, *CM* Community orientation, *To* Total Score, *Sa* Satisfaction, *Lo* Loyalty

*NA* Not Applicable

* *p*-value<0.05

** *p*-value<0.01

**Fig. 3:**
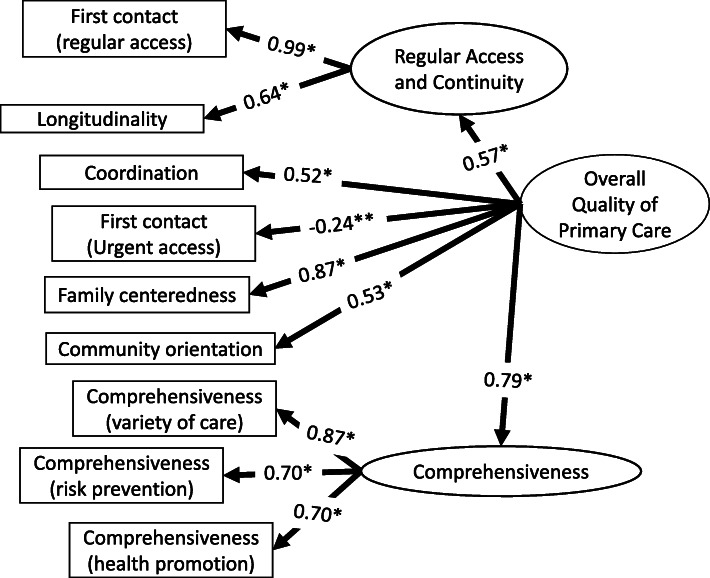
CFA model for overall quality of primary care. * *P* < 0.05. ** *P* < 0.01

Overall 118/206 questionnaires were analysed using SEM. The remaining 88 questionnaires were excluded as they contained at least one missing value across the nine component scales. A sample of more than 100 was considered as the minimum satisfactory sample size when conducting SEM [36]. The assumption of normality was met, although mild skewness or kurtosis was also confirmed (Table 3). In the first SEM phase, we conducted a confirmatory factor analysis (CFA) to assess the specified model based on the results of the preceding analyses and previous literature. When specifying the model, we added two latent variables to allow the combination of similar component scales: *comprehensiveness* and *regular access and continuity*. Comprehensiveness-related items, namely *comprehensiveness (various services)*, *comprehensiveness (risk prevention)*, and *comprehensiveness (health promotion)*, were combined into the *comprehensiveness* latent variable. *Access* was also noted as a dimension of *continuity,* [37] and several previous studies classified access to care and continuity of care into one component [6, 38]. *First contact (regular access)* and *longitudinality* were also classified into one component in EFA. *First contact (regular access)* and *longitudinality* were therefore combined to form a “*regular access and continuity*” latent variable. In addition, PCPs who provided each primary care element, placed “*overall quality of primary care*” as a concept encompassing all primary care functions. This model was identified by calculating degrees of freedom, and generalized least square estimator was selected for the estimation given that the smaller sample size performed better [34]. Modification was conducted during model evaluation, given that some variables were assumed to share error variance; *longitudinality* was considered to affect other primary care element through long standing relationship. Figure 3 shows the CFA model for overall quality of primary care (detailed description in Additional file 4). The robustness scale of the CFA model was shown by the RMSEA to be 0.000, with a 90% confidence interval (CI) of (0.000, 0.074), adjusted goodness-of-fit index (AGFI) as 0.917, and χ^2^ (22) = 21.3; *p* = 0.50.

**Fig. 4:**
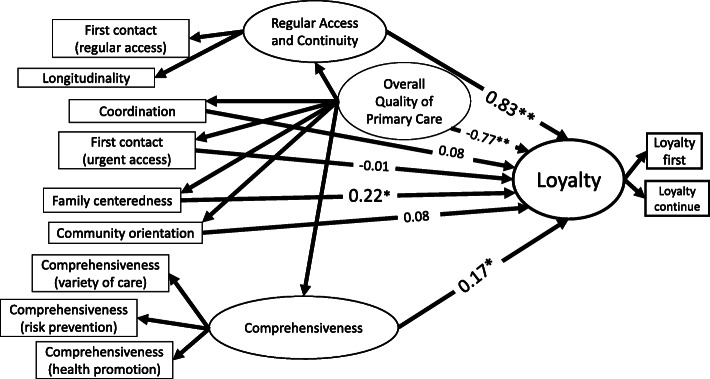
SEM analysis for the quality of primary care and patient loyalty. * *P* < 0.05. ** *P* < 0.01. Loyalty first: the willingness to go to your PCP with a new health problem. Loyalty continue: the willingness to continue seeing your PCP

We compared the PCAT to the satisfaction model derived from the preceding CFA model. Because each element was considered to affect patient satisfaction, paths were depicted to satisfaction. However, a model for patient satisfaction was not identified. Next, we compared the PCAT to the loyalty model derived from the preceding CFA model. A path was depicted to loyalty from each element, similar to the prior satisfaction model. When evaluating the model, modification was conducted because the element of loyalty was assumed to share error variance with “*regular access and continuity*”. Figure 4 shows SEM analysis for the quality of primary care and patient loyalty (detailed description in Additional file 5). For this model including loyalty, RMSEA was 0.019 with 90% CI of (0.000, 0.073), AGFI is 0.893, and χ^2^ (31) = 32.4; *p* = 0.39. *Regular access and continuity* and *family-centeredness* were significantly related to loyalty. The total effect (direct effect) of the overall quality of primary care was 0.098.

## Discussion

The main findings of this study were that *longitudinality*, *first contact (regular access)*, and *family-centeredness* were positively related to patient loyalty, or the willingness to revisit a particular primary healthcare provider.

We conducted this study in a rural area, allowing us to compare the results with those obtained in a previous Japanese PCAT study, [11] conducted in a metropolitan area. In the principal component analysis, *longitudinality*, *first contact (regular access)*, and *family-centeredness* were included in one component scale. Of these, *first contact (regular access)* and *family-centeredness* were not included in the previous study [11]. This may have been due to the different analysis methods employed in the aforementioned study due to the increased population density and high item-factor loading use (> 0.50) during item selection. Here, we considered those component scales to be essential primary care concepts; rural family physicians provide a broader scope of practice than urban ones in terms of comprehensiveness, [39–41] family structure differs between urban and rural areas, [41] and family-centeredness is an important primary care element [40–42].

Here, first contact was divided into regular access and urgent access, the latter being a unique primary care element. All scales were inter-correlated, except for *First contact (urgent access)*, which was related to patient satisfaction but not loyalty (Table 3). *First contact (urgent access)* is mainly composed of out-of-hours access to primary care in this study. There are several previous studies regarding out-of-hours primary care, which is considered a distinct key element in health care systems [43–46]. Several studies of out-of-hours primary care and patient satisfaction indicated a more specific situation including care quality, the means of consultation, and waiting time [44, 46]. More detailed elucidation of *First contact (urgent access)* is required for further interpretation.

**Fig. 5:**
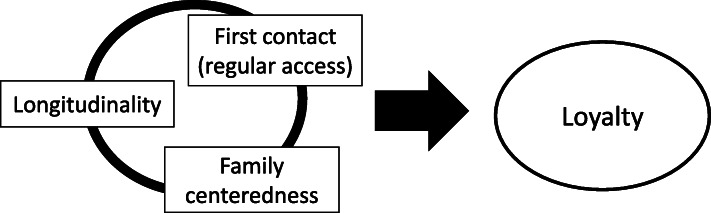
The concept that a greater understanding of the patient’s background and contextual circumstances contribute to patient loyalty

*Family-centeredness* was identified as an important function in primary care, although it is located as one of the supplementary elements compared with other core functions (first contact, longitudinality, comprehensiveness, and coordination) in PCAT [47]. *Family-centeredness* indicated the potential to improve patient and family satisfaction in a paediatrics study, more studies to measure the various outcomes in many fields are required [48, 49]. *Family-centeredness-*patient loyalty relationship, the question “Do you think your provider would meet with your family when you considered it to be necessary?” was highly related to patient loyalty. Additionally, *longitudinality*, *first contact (regular access)*, *family-centeredness* scales, and loyalty were highly inter-related. Thus, care practices based on a greater understanding of patients’ background and contextual circumstances, including family, improve patient loyalty (Fig. 5).

In terms of family-oriented primary care, the physician–patient–family relationship is an important treatment variable in fostering a patient’s positive health behaviours [42, 50]. Correspondingly, we revealed that *family-centeredness* was the variable strongly related to willingness to revisit one’s PCP.

It is important to consider the complexity of patients’ problems, as they often present with a combination of biological, psychological, family, and/or social problems. To treat these effectively, PCPs should have a deep understanding of their patients, including their medical conditions, various preferences, familial contexts, consultation accessibility, and methods of communication. The accumulation of these skills by PCPs is essential for cultivating patient loyalty, which is improved through consistent regular access and longitudinality.

This study has some limitations. The first relates to the selection biases. There is the potential there was an under-sampling of patients who were not satisfied with the quality of healthcare. Participant recruitment was carried out by the staff at each facility, and it is possible that they selected patients who they believed had a positive relationship with the facility. In addition, other selection biases exist for elderly and younger people. Frail elderly people who visit the primary care centre alone may not to have received the request to respond to this survey. We made particular effort to gather elderly respondents because they are the majority age group of these three regions. We advised research assistants to be sure to include elderly people who were able to respond with a companion such as a family member. Because the special training was not done for the research assistant, our study might include more active people, or elderly individuals who have family members who lived nearby. Although we conducted the survey in medical facilities, younger people go to these facilities less frequently than the elderly, thus, the number of younger people included in this study is relatively small. Secondly, we could not identify a patient satisfaction model through the SEM analysis. We had to take several error correlations into account to analyse the structural equation model (loyalty), as primary care elements were inter-correlated. Particularly, *longitudinality* and *first contact (regular access)* were significantly highly inter-correlated; we needed to arrange several error correlations for these analyses. Additionally, *patient loyalty* was closely aligned with *usual access* and *continuity*, perhaps due to the relatively small sample size and the model’s complicated nature, requiring further adjustment. Although SEM was an appropriate statistical method here (a high number of correlations disqualifies logistic regression analysis), further data should be obtained, or items should be modified, to reduce the number of error terms. Despite these limitations, this study has several strengths and unique contributions: the instrument we developed demonstrated high validity and reliability; the survey supported the association between primary care quality and patient quality compared with satisfaction; and a loyalty model for SEM analysis showed appropriate results.

## Conclusions

*Longitudinality*, *regular access*, and *family-centeredness* were correlated with each other, all of which cultivate patient loyalty. In their practice settings PCPs need to be mindful of longitudinal relationships with patients including their family contexts and the consistency of their regular access.

## Supplementary Information

**Additional file 1.** Comparison between reference population and respondents.

**Additional file 2.** Developed Questionnaire (in English) and correlations with patient satisfaction/loyalty.

**Additional file 3.** Developed Questionnaire (in Japanese).

**Additional file 4.** Detailed description of SEM analysis.

**Additional file 5.** Detailed description of SEM analysis for the primary care and patient loyalty.

## Data Availability

The datasets used and/or analysed in the current study are available from the corresponding author upon reasonable request.

## Abbreviations

AGFI: Adjusted Goodness-of-Fit Index
CFA: Confirmatory Factory Analysis
CI: Confidence Interval
EFA: Exploratory Factor Analysis
GPAS: General Practice Assessment Survey
GPAQ: General Practice Assessment Questionnaire
PCAT: Primary Care Assessment Tool
PCP: primary care provider
RMSEA: Root Mean Square Error of Approximation
SEM: Structural Equation Modelling
