# Supplementary material for: Patient satisfaction and loyalty in Japanese primary care: a cross-sectional study
Source: BMC Health Serv Res. 2021 Mar 25;21:274. doi: 10.1186/s12913-021-06276-9 (PMC7992825; doi:10.1186/s12913-021-06276-9)

Additional File 1: Comparison between reference population and respondents

Population and years in A city

Gender: Male 47%（≧20 year-old）

Respondents of our study in A city (n=48, 1 clinic)

Gender: Male 18%（≧20 year-old）


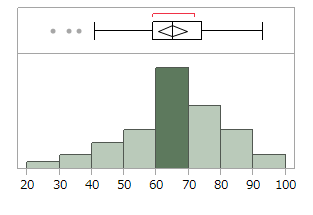


Population and years in B city

Gender：Male 41%（≧20 year-old）

Respondents of our study in B city (n=108, 3 clinics)

Gender: Male 42%


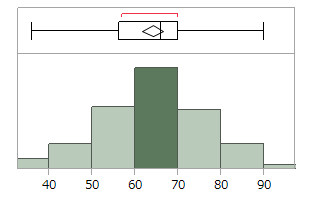


Population and years in C town

Gender: Male 49%（≧20 year-old）

Respondents of our study in C town (n= 50, 1 clinic)

Gender: Male 20％


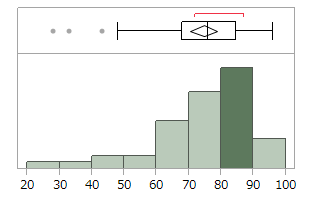

Supplement: Supplementary file 1 — Additional file 1. Comparison between reference population and respondents. [file 12913_2021_6276_MOESM1_ESM.docx]
