# Supplementary material for: Patient satisfaction and loyalty in Japanese primary care: a cross-sectional study
Source: BMC Health Serv Res. 2021 Mar 25;21:274. doi: 10.1186/s12913-021-06276-9 (PMC7992825; doi:10.1186/s12913-021-06276-9)
Supplement: Supplementary file 2 — Additional file 2. Developed Questionnaire (in English) and correlations with patient satisfaction/loyalty. [file 12913_2021_6276_MOESM2_ESM.docx]

| Additional File 2. Developed Questionnaire (in English) and correlations with patient satisfaction/loyalty | | | | | |
| --- | --- | --- | --- | --- | --- |
| Scale | Item codes | Developed questionnaire (in English) | Expert round (median) | Correlation^†^ with satisfaction | Correlation^†^ with loyalty |
| First contact  (regular access)^‡^ | B1 | If you become ill during office hours, can your provider see you on that day? | 8 | 0.20* | 0.40* |
|  | B2 | If you call during office hours, can you quickly get advice over the phone? | 8 | 0.23* | 0.46* |
|  | B3 | Can you easily get a physical examination (check-up) from your provider? | 9 | 0.18^††^ | 0.39* |
|  | B4 | Can you see your provider any time you like? | 8 | 0.15^††^ | 0.38* |
|  | B5 | Is your provider’s location (building) easy to access? (for example, barrier-free entrance and rooms, western toilet, etc.) | 8.5 | 0.21* | 0.49* |
| First contact  (urgent access) | C1 | If you become ill outside of office hours, can you consult with your provider over the phone? | 8 | 0.20^*^ | -0.04 |
|  | C2 | If you become ill outside of office hours, at night or on a holiday, can you see your provider that day (or night)? | 8 | 0.21^*^ | -0.04 |
|  | C3 | When you go to your provider, do you wait more than 30 minutes? | 8 | 0.29^*^ | 0.02 |
| Longitudinality^‡^ | D1 | When visiting your provider, do you see the same doctor each time? | 8 | 0.13 | 0.47* |
|  | D2 | Do you think your provider accurately understands what you say and ask? | 8.5 | 0.39^*^ | 0.69* |
|  | D3 | When answering your questions, does your provider answer them in a way that you can understand? | 9 | 0.40^*^ | 0.61* |
|  | D4 | Can you ask a doctor or nurse, who knows you well, questions over the phone? | 7.5 | 0.24^*^ | 0.37* |
|  | D5 | Does your provider take enough time to discuss your concerns and problems? | 8 | 0.22^*^ | 0.46* |
|  | D6 | Do you feel comfortable talking about your concerns and problems with your provider? | 9 | 0.28^*^ | 0.54* |
|  | D7 | Does your provider understand, not just your illnesses, but also you as a person? | 8.5 | 0.28^*^ | 0.52* |
|  | D8 | Does your provider know who is living with you? | 9 | 0.18^††^ | 0.50* |
|  | D9 | Does your provider know what issue is the most important to you? | 9 | 0.25^*^ | 0.50* |
|  | D10 | Does your provider know about your previous illnesses? | 8 | 0.24^*^ | 0.59* |
|  | D11 | Does your provider know about your job and employment status? | 8 | 0.26^*^ | 0.53* |
|  | D12 | Does your provider know if you have had trouble getting or paying for medicines you needed? | 7^§^ | 0.35^*^ | 0.26* |
|  | D13 | Does your provider know about all the medications you are currently taking? | 9 | 0.18^††^ | 0.60* |
|  | D14 | Does your provider explain your test results to you? | 7.5 | 0.28^*^ | 0.45* |
|  | D15 | When you see your provider, do you bring your medical records with you? (for example, medication booklet, blood pressure diary, blood test results, vaccination records, etc.) | 7.5 | 0.26^*^ | 0.40* |
| Coordination | E1 | Did your provider recommend that you consult another medical facility in order to solve your health problems? | 9 | 0.28* | 0.39* |
|  | E2 | Did your provider adequately explain the reasons for referring you to a specialist in a note to the specialist? | 9 | 0.22* | 0.41* |
|  | E3 | Did your provider adequately explain the reasons for referring you to a specialist in a note to the specialist? | 8.5 | 0.30* | 0.36* |
|  | E4 | Does your provider know the results of your consultation with the specialist? | 9 | 0.30* | 0.45* |
|  | E5 | After your consultation with the specialist, did your provider ask you about the kind of treatment you received there? | 8 | 0.26* | 0.44* |
|  | E6 | Was your provider interested in the quality of care you received from the specialist you consulted? | 7.5 | 0.25* | 0.55* |
| Comprehensiveness (variety of care) | F1 | Mental health counselling | 8 | 0.34* | 0.31* |
|  | F2 | Hearing testing | 8.5 | 0.21* | 0.08 |
|  | F3 | Vision testing | 8.5 | 0.22* | 0.07 |
|  | F4 | Smoking cessation (consultation about quitting smoking) | 8.5 | 0.28* | 0.08 |
|  | F5 | Removal and treatment of warts | 8 | 0.08 | 0.22* |
|  | F6 | Care of ingrown toenails | 8.5 | 0.04 | 0.17^††^ |
|  | F7 | Suturing of wounds | 8.5 | 0.05 | 0.27* |
|  | F8 | First aid treatment of ankle sprains (applying a support bandage or splint) | 8 | 0.10 | 0.26* |
|  | F9 | Consultation on joint or muscle pain in the lower back, shoulders, or knees | NA^\|\|^ | 0.14 | 0.44* |
|  | F10 | Consultation on costs of the social security system and welfare benefits your family can receive (enquiry) | 7.5 | 0.33* | 0.12 |
|  | F11 | Consultation on how to deal with a family member who can no longer make their own healthcare decisions | 8 | 0.15 | 0.35* |
|  | F12 | Family consultation on use of nursing homes and long-term care insurance | 8 | 0.10 | 0.27* |
|  | F13 | Family planning and birth control methods | 9 | 0.17 | 0.10 |
|  | F14 | Cervical cancer testing | 7.5 | 0.16 | 0.18 |
|  | F15 | Consultation on problems related to pregnancy | 9 | 0.24^††^ | 0.15 |
| Comprehensiveness (risk prevention) | G1 | Advice on how to use a seatbelt or child seat | 8 | 0.31* | 0.10 |
|  | G2 | How to deal with troubles in the home environment (child-rearing, sharing duties of long-term care, violence, etc.) | 8 | 0.02 | 0.23* |
|  | G3 | Potential exposure to harmful substances in the home environment, workplace, and community | 8.5 | 0.25* | 0.11 |
|  | G4 | Prevention of hot water burns | 8.5 | 0.09 | 0.06 |
|  | G5 | Prevention of falls | 8.5 | 0.03 | 0.32* |
|  | G6 | For women: prevention of osteoporosis and bone weakening | 8.5 | 0.03 | 0.26* |
|  | G7 | For women: care for problems related to normal menstruation and menopause | 8 | 0.08 | 0.23^††^ |
| Comprehensiveness (health promotion) | H1 | Answer questions about nutrition and healthy diet (explanation) | 8 | 0.17^††^ | 0.36* |
|  | H2 | Immunisations (shots) | 9 | 0.19^††^ | 0.32* |
|  | H3 | Advice on healthy and unhealthy foods, and on getting a good night’s sleep | 8 | 0.19^††^ | 0.31* |
|  | H4 | Advice about appropriate exercise | 8 | 0.22* | 0.41* |
|  | H5 | Review and advice on oral medications and supplements being taken | 9 | 0.34* | 0.09 |
| Family-centeredness^‡^ | I1 | When planning treatment and care for you or your family, does your provider ask for your thoughts and opinions? | 9 | 0.22* | 0.44* |
|  | I2 | Do you think your provider would meet with your family when you considered it necessary? | 8 | 0.26* | 0.51* |
| Community orientation | J1 | Is your provider aware of important health problems in the area where you live? | 8 | 0.17^††^ | 0.21* |
|  | J2 | Does your PCP get opinions and ideas from people (including residents and professionals other than medical staff) that will help to provide better health care? | 8 | 0.26* | 0.24* |
| NA: Not applicable | | | |  |  |
| * p-value ＜ 0.01 | | | | | |
| ^†^Spearman’s correlation | | | | | |
| ^††^ p-value ＜ 0.05 | | | | | |
| ^§^ This item was deleted at the first-round expert review. However, it was decided to reintroduce it in the final review discussion. | | | | | |
| ^\|\|^ This item was added following the non-medical expert review and final review. | | | |  |  |

^‡^ First contact (regular access), Longitudinality, and Family-centeredness was divided one of the seven components in the initial principal component analysis.
