# Supplementary material for: Patient satisfaction and loyalty in Japanese primary care: a cross-sectional study
Source: BMC Health Serv Res. 2021 Mar 25;21:274. doi: 10.1186/s12913-021-06276-9 (PMC7992825; doi:10.1186/s12913-021-06276-9)
Supplement: Supplementary file 3 — Additional file 3. Developed Questionnaire (in Japanese). [file 12913_2021_6276_MOESM3_ESM.docx]

| Additional File 3. Developed Questionnaire (in Japanese) | | |
| --- | --- | --- |
| Scale | Item codes | Developed Questionnaire (in Japanese) |
| First contact  (regular access) | B1 | かかりつけの診療時間**内**に具合が悪くなった場合、その日のうちに診察してもらえますか？ |
|  | B2 | かかりつけの診療時間**内**であれば、電話で迅速にアドバイスをもらうことができますか？ |
|  | B3 | かかりつけで健康診断を受けることは、容易にできますか？ |
|  | B4 | 受診したいと思うとき、かかりつけはいつでも診てくれますか？ |
|  | B5 | かかりつけの建物は、使いやすい構造になっていますか？（入口や屋内のバリアフリーの状態、洋式トイレの設置など） |
| First contact  (urgent access) | C1 | かかりつけの診療時間外に具合が悪くなった場合、診療時間外でも電話で相談をすることができますか？ |
|  | C2 | かかりつけの診療時間外で、夜間や休日等に具合が悪くなった場合、その日（夜）のうちに診察してもらえますか？ |
|  | C3 | かかりつけでの待ち時間は、３０分未満ですか？ |
| Longitudinality | D1 | かかりつけに行くとき、毎回**同じ**医師に診てもらえますか？ |
|  | D2 | かかりつけは、**あなたの発言や質問を正確に理解している**と思いますか？ |
|  | D3 | かかりつけはあなたの質問に答えるとき、**理解できるように答えて**くれていますか？ |
|  | D4 | あなたのことをよく知る医師や看護師に電話で質問することができますか？ |
|  | D5 | かかりつけは、あなたの心配事や問題について話す時間を十分にとってくれますか？ |
|  | D6 | 心配事や問題について、かかりつけに気軽に話すことができますか？ |
|  | D7 | かかりつけは、病気だけでなくあなた自身についてもよく理解してくれていますか？ |
|  | D8 | かかりつけは、 あなたが誰と同居しているか知っていますか？ |
|  | D9 | かかりつけは、あなたの１番重要な問題が何か分かっていますか？ |
|  | D10 | かかりつけは、あなたのこれまでの病気の経過を知っていますか？ |
|  | D11 | かかりつけは、あなたの仕事や雇用の状況を知っていますか？ |
|  | D12 | あなたのかかりつけは、医療費の支払いについて困っているかどうか知っていますか？ |
|  | D13 | かかりつけは、あなたが服用している薬剤をすべて知っていますか？ |
|  | D14 | かかりつけで、ご自分の検査結果について説明を受けていますか？ |
|  | D15 | かかりつけを受診するとき、何らかの自分の診療記録（お薬手帳、血圧手帳、採血結果や予防注射歴など）を持っていきますか？ |
| Coordination | E1 | かかりつけは、あなたの抱える健康問題を解決するために、他の医療機関の受診について相談してくれましたか？ |
|  | E2 | かかりつけまたはその施設のスタッフは、他の医療施設を受診するために予約を取ってくれましたか？ |
|  | E3 | かかりつけは、専門医あての紹介状に受診理由などについて十分に記載してくれましたか？ |
|  | E4 | かかりつけは専門医受診の結果を知っていますか？ |
|  | E5 | 専門医または専門の施設を受診した後、かかりつけはそこで受けた診療内容についてあなたにたずねましたか？ |
|  | E6 | かかりつけは、あなたが専門医または専門の施設で受けた医療の質に関心を持っているようですか？ |
| Comprehensiveness (variety of care) | F1 | 精神的な問題の相談 |
|  | F2 | 聴力検査 |
|  | F3 | 視力検査 |
|  | F4 | 禁煙外来（禁煙についての相談） |
|  | F5 | 疣（いぼ）の処置・除去 |
|  | F6 | 陥入爪（足の巻き爪）のケア |
|  | F7 | 傷口の縫合 |
|  | F8 | 足首捻挫の応急処置（サポーターや副木による固定） |
|  | F9 | 腰痛、膝または肩が痛いといった関節や筋肉の痛みの相談 |
|  | F10 | ご家族が受けられる社会保障制度や福祉手当の有無についての相談（問い合わせ） |
|  | F11 | 家族の誰かが治療に対する意思決定ができなくなった場合の対処方法の相談 |
|  | F12 | 老人ホームなどの施設入所や介護保険の利用についての家族への提案や相談 |
|  | F13 | 家族計画法や避妊法 |
|  | F14 | 子宮頸がん検査 |
|  | F15 | 妊娠に伴う問題についての相談 |
| Comprehensiveness (risk prevention) | G1 | シートベルトやチャイルドシートの使用についての助言 |
|  | G2 | 家庭内のもめごと（子育て、介護の分担、暴力など）への対処方法 |
|  | G3 | 自宅や職場、地域で有害物質にさらされる可能性 |
|  | G4 | 熱湯やけどの予防対策 |
|  | G5 | 転倒の予防対策 |
|  | G6 | **女性に対して**：骨粗鬆症や骨脆弱化（骨が弱くなること）の予防対策 |
|  | G7 | **女性に対して**：通常の月経や更年期に伴う問題のケア |
| Comprehensiveness (health promotion) | H1 | 栄養や食事療法についての質問に対する回答（説明） |
|  | H2 | 予防接種（注射） |
|  | H3 | 健康によい食品や悪い食品について、 あるいは十分な睡眠をとることについての助言 |
|  | H4 | 適切な運動についての助言 |
|  | H5 | 内服中の薬やサプリメントについての確認や相談 |
| Family centeredness | I1 | あなたやあなたの家族の治療・ケアを計画するとき、かかりつけは**あなたの**考えや意見を尋ねますか？ |
|  | I2 | かかりつけは、あなたが必要だと思ったときにあなたの家族と面会してくれると思いますか？ |
| Community orientation | J1 | かかりつけは、あなたが住む地域の重要な健康問題について知っていますか？ |
|  | J2 | かかりつけは、よりよい医療を提供するために、人（住民や医療者以外の職種も含めて）の意見や考えを聞いていますか？ |
| Loyalty | |  |
| The willingness to continuity | | 今後も継続して、今のかかりつけに診てもらいたいと思いますか？ |
| the willingness to first contact | | 新たな健康問題が生じたとき、まずあなたのかかりつけに行きますか？ |
