# Supplementary figures and images for: Patient satisfaction and loyalty in Japanese primary care: a cross-sectional study
Source: BMC Health Serv Res. 2021 Mar 25;21:274. doi: 10.1186/s12913-021-06276-9 (PMC7992825; doi:10.1186/s12913-021-06276-9)

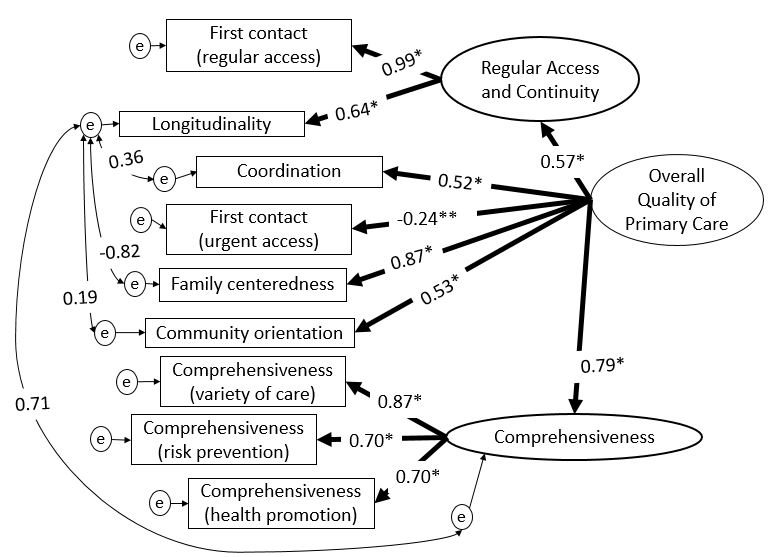


Additional File4. Detailed description of SEM analysis

* *P* < 0.05, ** *P* < 0.01.

Supplement: Supplementary file 4 — Additional file 4. Detailed description of SEM analysis. [file 12913_2021_6276_MOESM4_ESM.docx]
