# Supplementary material for: Patient satisfaction and loyalty in Japanese primary care: a cross-sectional study
Source: BMC Health Serv Res. 2021 Mar 25;21:274. doi: 10.1186/s12913-021-06276-9 (PMC7992825; doi:10.1186/s12913-021-06276-9)
Supplement: Supplementary file 5 — Additional file 5. Detailed description of SEM analysis for the primary care and patient loyalty. [file 12913_2021_6276_MOESM5_ESM.docx]

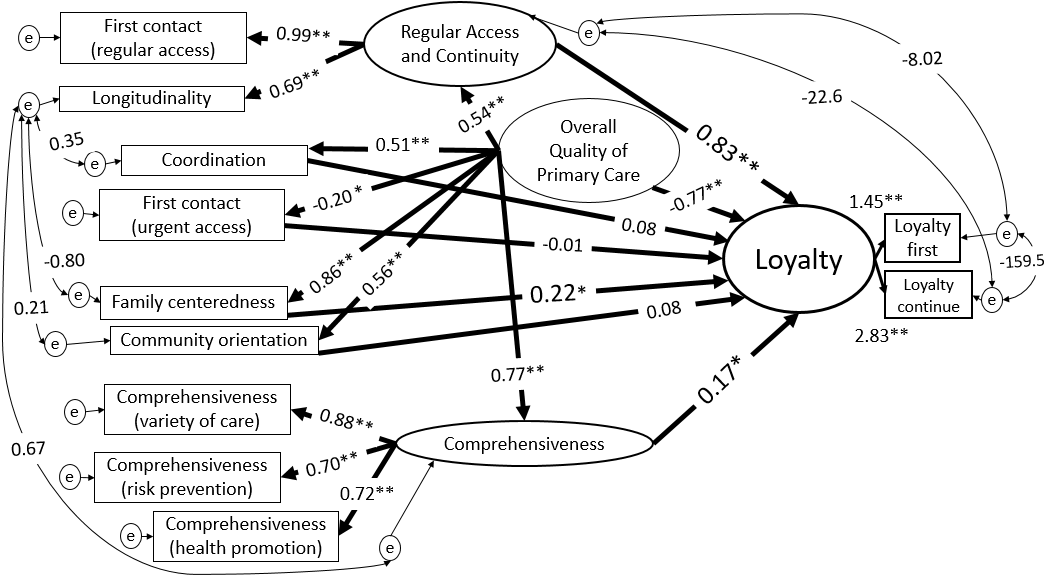


Additional File 5. Detailed description of SEM analysis for the quality of primary care and patient loyalty.

* *P* < 0.05, ** *P* < 0.01.
